# Quantitative Data From Rating Scales: An Epistemological and Methodological Enquiry

**DOI:** 10.3389/fpsyg.2018.02599

**Published:** 2018-12-21

**Authors:** Jana Uher

**Affiliations:** London School of Economics and Political Science, London, United Kingdom

**Keywords:** qualitative-quantitative integration, observational methods, assessment methods, transdisciplinary approach, quantitative methods in the social sciences, measurement, quantification, data

## Abstract

Rating scales are popular methods for generating quantitative data directly by persons rather than automated technologies. But scholars increasingly challenge their foundations. This article contributes epistemological and methodological analyses of the processes involved in person-generated quantification. They are crucial for measurement because data analyses can reveal information about study phenomena only if relevant properties were encoded systematically in the data. The Transdisciplinary Philosophy-of-Science Paradigm for Research on Individuals (TPS-Paradigm) is applied to explore psychological and social-science concepts of measurement and quantification, including representational measurement theory, psychometric theories and their precursors in psychophysics. These are compared to theories from metrology specifying object-dependence of measurement processes and subject-independence of outcomes as key criteria, which allow tracing data to the instances measured and the ways they were quantified. Separate histories notwithstanding, the article’s basic premise is that general principles of scientific measurement and quantification should apply to all sciences. It elaborates principles by which these metrological criteria can be implemented also in psychology and social sciences, while considering their research objects’ peculiarities. Application of these principles is illustrated by quantifications of individual-specific behaviors (‘personality’). The demands rating methods impose on data-generating persons are deconstructed and compared with the demands involved in other quantitative methods (e.g., ethological observations). These analyses highlight problematic requirements for raters. Rating methods sufficiently specify neither the empirical study phenomena nor the symbolic systems used as data nor rules of assignment between them. Instead, pronounced individual differences in raters’ interpretation and use of items and scales indicate considerable subjectivity in data generation. Together with recoding scale categories into numbers, this introduces a twofold break in the traceability of rating data, compromising interpretability of findings. These insights question common reliability and validity concepts for ratings and provide novel explanations for replicability problems. Specifically, rating methods standardize only data formats but not the actual data generation. Measurement requires data generation processes to be adapted to the study phenomena’s properties and the measurement-executing persons’ abilities and interpretations, rather than to numerical outcome formats facilitating statistical analyses. Researchers must finally investigate how people actually generate ratings to specify the representational systems underlying rating data.

## Introduction

Quantifications are central to many fields of research and applied settings because numerical data allow to analyze information using the power of mathematics (Chalmers, 2013; Porter, 1995; Trierweiler and Stricker, 1998). In psychology and social sciences (e.g., education, sociology, political science), quantitative data are often generated with rating methods in which persons indicate their judgments of predefined statements on multi-stage scales (e.g., standardized assessments, surveys or questionnaires). Rating scales are also used in many applied sectors (e.g., government, business, management, industry, public media) to help answer key questions, make decisions and develop strategies, such as for national policies, health programs, personnel selection and marketing (Menon and Yorkston, 2000; Abran et al., 2012; Hammersley, 2013). Accurate quantifications are thus critically important.

### Increasing Criticism of Rating Scales

The strong reliance on rating methods is, however, increasingly criticized (Baumeister et al., 2007; Fahrenberg et al., 2007; Grzyb, 2016; Doliński, 2018). Scholars from various disciplines scrutinize their underlying epistemologies and measurement theories (Wagoner and Valsiner, 2005; Trendler, 2009; Vautier et al., 2012; Hammersley, 2013; Bringmann and Eronen, 2015; Buntins et al., 2016; Tafreshi et al., 2016; Bruschi, 2017; Humphry, 2017; Valsiner, 2017; Guyon et al., 2018). These developments are still largely unnoticed by mainstream psychologists who currently focus on the replication crisis, which they aim to solve by scrutinizing the epistemological foundations of significance testing, confidence interval estimations and Bayesian approaches (Nosek et al., 2015; Open Science Collaboration, 2015; Wagenmakers et al., 2016; Zwaan et al., 2017)—thus, by improving issues of data *analysis*.

But processes of data *generation* are largely understudied. Discussions are limited to debates about so-called ‘qualitative’ versus ‘quantitative’ methods, a common polarization suggesting some methods could be quantitative but not qualitative, and vice versa. Previous debates revolve around irreconcilable differences in underlying epistemologies (e.g., constructivist versus naïve-realist). To balance their respective advantages and disadvantages, both methods are combined in mixed-method designs (Creswell, 2003). But the methodological foundations of the operational procedures by which ‘quantitative’ and ‘qualitative’ data are generated are hardly discussed. Specifically, so-called ‘quantitative’ data are commonly generated by lay people who may be largely unaware of the positivist epistemology underlying the scales they are ticking. But even if they knew, what would this tell them about how to generate data? Likewise, laypeople are commonly unfamiliar with measurement theories. So how can they, by intuitively judging and ticking scales, produce data that meet the axioms of quantity and measurement? What considerations and decisions must raters actually make to justify interpretation of rating outcomes as ‘quantitative’ data? And in what ways do scientists’ axioms and theories of measurement inform raters’ decisions? Current debates are surprisingly silent about these fundamental issues.

Problematic findings with rating scales increasingly emerge. On widely used Big Five personality scales, differences between student and general public samples varied substantially and randomly across 59 countries, showing that, contrary to common assumptions, student findings cannot be generalized (Hanel and Vione, 2016). The empirical interrelations among ratings items used to assess the same personality factor (e.g., ‘outgoing’ and ‘not reserved’ for Extraversion) varied unsystematically across 25 countries, averaging around zero (Ludeke and Larsen, 2017). These findings seriously question what information these ratings actually capture.

For wide applications, rating scales are worded in everyday language, thus capitalizing on raters’ and scientists’ everyday knowledge. But everyday knowledge is often incoherent, contradictive and context-dependent (Laucken, 1974; Hammersley, 2013). What specific knowledge do raters actually apply? Could it be that ‘outgoing’ has not the same meaning for students and the general public and not the same for people from different countries? How do raters choose the scale categories to indicate their judgements? What does “agree” actually mean to different people and in what ways is this related to their intuitive judgements and scientists’ axioms of quantity? Rating data have been used intensely for almost a century now (Thurstone, 1928; Likert, 1932); but still little is known about the processes by which raters actually generate these data.

### Aims of This Article

This article contributes to current debates an enquiry of the epistemological and methodological foundations of rating scales, which psychologists and social scientists widely use to generate quantitative data *directly by persons* rather than using technologies (see concepts of ‘persons as data generation systems’, ‘human-based measurement’, ‘measurement with persons^1^’, ‘humans as measurement instrument’; Berglund, 2012; Pendrill, 2014). The focus is on intuitive judgements on multi-stage rating scales (e.g., Likert-style), not considering comparative judgment methods (Thurstone, 1927) or questionnaires involving right and wrong answers (e.g., intelligence tests). The article explores *processes of data generation*—before any methods of data *analysis* can be applied. These processes are crucial for measurement and quantification because data can reveal information about study phenomena *only if* relevant properties have been encoded systematically in the data. No method of analysis, however, sophisticated, can substitute these essential steps.

A transdisciplinary perspective is adopted to elaborate epistemological, metatheoretical and methodological foundations of theories and methods of data generation, measurement and quantification from psychology and social sciences but also from biology, physics and especially *metrology*, the science of measurement (BIPM, 2006). Metrology was key to the successes of the physical sciences (e.g., physics, chemistry, astronomy) but did not form the basis for measurement theories in psychology and social sciences (Michell, 1999; Mari, 2013). This notwithstanding, the article’s basic premise is that *general principles of scientific measurement and quantification should apply to all sciences* (see also McGrane, 2015; Mari et al., 2017). This is no utopic ideal. It is a necessity arising from the complexity of today’s real-world problems that require application of inter-, multi- and transdisciplinary approaches. Big Data gain momentum. But statistical results can be interpreted with regard to real-world phenomena *only if* the data fulfill elementary criteria of measurement and quantification that can be understood and used in the same way across sciences—without ignoring peculiarities of their objects of research.

Psychologists and social scientists encounter particular challenges because their study phenomena are intangible, highly adaptive and complex, and less rigorously rule-bound than those explored in other fields (but see Hossenfelder, 2018). Therefore, measurement technologies from physical sciences and engineering cannot be applied. Moreover, as all persons are individuals and members of social communities, scientists exploring these phenomena cannot be independent of their objects of research. This entails particular risks of (unintentionally) introducing all kinds of ego-centric and ethno-centric biases (Uher et al., 2013b; Uher, 2015c).

To elaborate principles by which basic criteria of measurement and quantification can be met in all sciences while considering fundamental differences in their objects of research, this article applies the *Transdisciplinary Philosophy-of-Science Paradigm for Research on Individuals* (*TPS-Paradigm*; Uher, 2015a,b,c,d,e, 2016a,b, 2018b,c). It is well suited for this purpose because it provides unitary frameworks in which concepts from psychology, life sciences, social sciences, physical sciences and metrology that are relevant for research on individuals have been systematically integrated. It also puts into focus the individuals who are doing research and generating data, thus opening up a meta-perspective on research processes.

First, these frameworks and relevant concepts are briefly introduced and used to explore epistemological foundations of measurement and quantification considering concepts from psychology, social sciences and metrology. Then, principles by which metrological criteria can also be met in person-generated quantifications are outlined, highlighting challenges and limitations. Application of these principles is illustrated by the example of investigations of individual-specific behaviors (‘personality’). The demands that rating methods impose on data-generating persons are systematically deconstructed and compared with the demands involved in other quantitative methods (e.g., ethological observations). Closing, the article highlights problematic assumptions underlying rating methods as well as implications for their utility to improve replicability and transparency in psychology and social sciences.

## Transdisciplinary Philosophy-Of-Science Paradigm for Research on Individuals (TPS-Paradigm)

**FIGURE 1:**
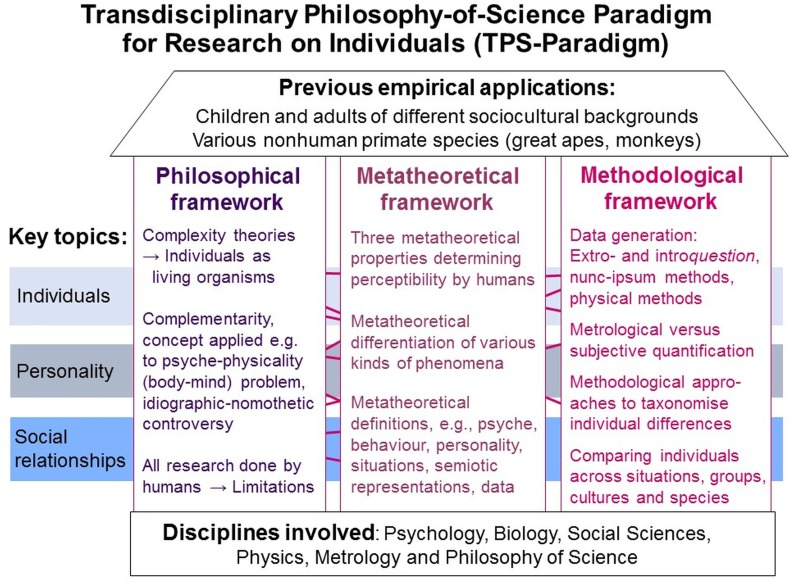
TPS-Paradigm: schematic overview. Its interrelated frameworks and key topics, the disciplines involved and previous applications in empirical studies.

The TPS-Paradigm comprises a system of interrelated philosophical, metatheoretical and methodological frameworks (*paradigm*) in which concepts, approaches and methods from various disciplines (*transdisciplinary*) for exploring phenomena in or in relation to *individuals* were systematically integrated, further developed and complemented by novel ones. Its purpose is to make explicit the presuppositions, metatheories and methodologies underlying scientific systems (*philosophy-of-science*) to help researchers critically reflect on; discuss and refine their theories, models and practices; and derive ideas for novel developments (for a schematic overview, see Figure 1; for introductions Uher, 2015a,c, 2018c; for more information and empirical applications^2^).

### Philosophical Framework

The philosophical framework specifies presuppositions made about individuals’ nature and properties and the fundamental notions by which knowledge about them can be gained. Three presuppositions are important.

#### Complexity Theories

Complexity theories, developed amongst others in philosophy (Hartmann, 1964), thermodynamics (Prigogine and Stengers, 1984), physics of life (Capra, 1997), theoretical biology (von Bertalanffy, 1937), medicine (Rothschuh, 1963), and psychology (Wundt, 1863; Koffka, 1935; Vygotsky and Luria, 1994) allow to conceive individuals as living organisms organized at different levels forming nested systems, from molecules and cells over individuals up to societies. At each level, they function as integrated wholes in which dynamic non-linear processes occur from which new properties emerge not completely predictable from their constituents (*principle of emergence*). These new properties can feed back to the constituents from which they emerge, causing complex patterns of upward and downward causation. With increasing levels of organization, ever more complex systems and phenomena emerge that are less rule-bound, highly adaptive and historically unique (Morin, 2008). This applies especially to psychological and social-science objects of research.

#### Complementarity

This concept highlights that particular objects of research can be exhaustively understood only by describing two mutually exclusive properties that are irreducible and maximally incompatible with one another, thus requiring different frames of reference, truth criteria and investigative methods, and that may therefore be regarded as *complementary* to one another (Fahrenberg, 1979, 2013; Hoche, 2008; Walach, 2013). This concept was applied to the wave-particle dilemma in research on the nature of light (Heisenberg, 1927; Bohr, 1937) and to the body-mind problem (Brody and Oppenheim, 1969; Fahrenberg, 1979, 2013; Walach and Römer, 2011). In this problem, called *psyche-physicality problem* in the TPS-Paradigm given its particular terminology (see below; Uher, 2015c), complementarity takes a metaphysically neutral stance without making assumptions of either ontological dualism or monism while emphasizing the necessity for *methodical dualism* to account for observations of two categorically different realities that require different frames of reference, approaches and methods (Walach, 2013). In the TPS-Paradigm, complementarity is also applied to resolve the nomothetic-idiographic controversy in ‘personality’ research (Uher, 2015d).

#### Human-Made Science

The third presupposition concerns explicit recognition that *all science is created by humans*, hence on the basis of humans’ perceptual (Wundt, 1907) and conceptual abilities (interpretations; Peirce, 1958, CP 2.308). This does not imply ideas of radical constructivism (von Glasersfeld, 1991), positing that concepts had no representational connection with a reality existing outside of individuals’ minds and that knowledge could be developed without reference to an ontological reality in which humans have evolved over millions of years (Uher, 2015a). But it also clearly rejects naïve realist assumptions that individuals’ senses could enable direct and objective perceptions of the external reality ‘as it really is’. Instead, it highlights that we can gain access to this reality only through our human perceptual and cognitive abilities, which inevitably limits our possibilities to explore and understand this reality. This epistemological position comes close to those of critical realism (Bhaskar and Danermark, 2006) and pragmatism-realism (Guyon et al., 2018). They emphasize the reality of the objects of research and their knowability but also that our knowledge about this reality is created on the basis of our practical engagement with and collective appraisal of that reality. Knowledge is therefore theory-laden, socially embedded and historically contingent.

As science inherently involves an anthropocentric perspective, a *phenomenon* is defined in the TPS-Paradigm as anything that humans can perceive or (technically) make perceivable and/or that humans can conceive (Uher, 2015c). This notion differs from various philosophical definitions (e.g., Kant’s, 1781/1998).

### Metatheoretical Framework

#### Three Metatheoretical Properties Determining Perceptibility by Humans

The TPS-Paradigm’s metatheoretical framework builds on *three metatheoretical properties* conceivable in different forms for phenomena studied in research on individuals. These particular properties are considered because they determine a phenomenon’s perceptibility, which has important methodological implications (see below). Given the focus on research on individuals, these properties are conceived in dimensions of everyday experiences (e.g., scaled to human bodies, international time standards), ignoring micro- or macro-dimensions explored in some fields (e.g., atomic and outer-space dimensions).

These properties are (1) a phenomenon’s location in relation to the studied individual’s body (e.g., internal, external), (2) its temporal extension (e.g., transient, temporally extended)—both dimensional properties—and (3) its spatial extension conceived as physical versus “non-physical”. Physicality here refers to concepts of classical physics, because they match everyday experiences, unlike quantum physical ones. Physical denotes corporeal/bodily/material phenomena (matter) as well as immaterial physical phenomena (e.g., heat, movements), which are not corporeal in themselves but become manifest in material phenomena with which they are systematically connected. All physical phenomena are spatially extended. But spatial properties cannot be conceived for “non-physical” phenomena, which are not simply contrasted against the physical (as indicated by the quotation marks) and therefore conceived as complementary. This distinction resembles Descartes’ res extensa and res cogitans (Hirschberger, 1980) but implies only a methodical rather than an ontological dualism (Uher, 2015c, 2016a). These properties are labeled metatheoretical because they reflect a level of abstraction not commonly considered, and only time and space constitute ontological categories.

#### Different Kinds of Phenomena Studied in Research on Individuals

**FIGURE 2:**
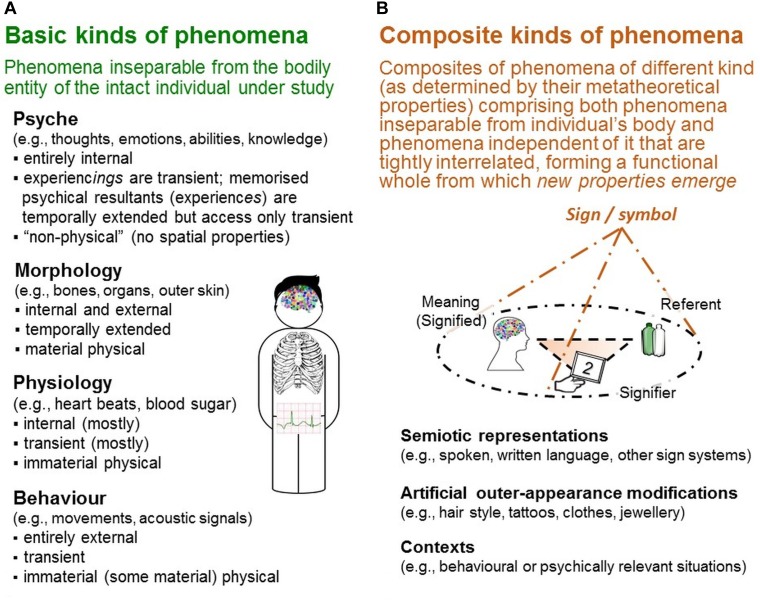
Kinds of phenomena. In the TPS-Paradigm, various kinds of phenomena are conceptually differentiated by the particular constellation of forms regarding the three metatheoretical properties determinating their perceptibility. Two types are distinguished: **(A)** basic kinds of phenomena are characterized by their inseparability from individuals’ bodies, and **(B)** composite kinds of phenomena by their complexity and heterogeneity of the phenomena involved, some of which are independent of individuals’ bodies.

The three properties are used to metatheoretically differentiate various *kinds of phenomena*, which differ in their particular constellations in these properties’ forms. For example, morphological phenomena (living organism’s structures and their constituting parts) are internal/external, temporally extended and material physical. Physiological phenomena (morphology’s chemical and physical functioning) are primarily internal, mostly transient and immaterial physical (Figure 2A). These *conceptual* differentiations, as they are accessibility-based, have important methodical implications for data generation shown below.

##### Basic kinds of phenomena: inseparable from the individual’s bodily entity

Four kinds of phenomena are conceived as *basic* because they are inseparable from the intact individual’s body: morphology, physiology, behavior and psyche (see Figure 2A; for details, Uher, 2015a). For the present analyses, the conceptual distinction between psyche and behavior is important.

*Behaviors* are defined as the “external changes or activities of living organisms that are functionally mediated by other external phenomena in the present moment” (Uher, 2016b, p. 490). Thus, behaviors are external, transient and (mostly immaterial) physical phenomena (e.g., movements, vocalizations). The *psyche* is defined as “the entirety of the phenomena of the immediate experiential reality both conscious and non-conscious of living organisms” (Uher, 2016a, p. 303; with immediacy indicating absence of phenomena mediating their perception; see Wundt, 1894). The psyche’s phenomena are essential for all sciences because they are the means by which any science is made. A science exploring the psyche must therefore distinguish between its objects of research and its tools for investigating them. Therefore, the psyche’s phenomena in themselves are termed *psychical*, whereas *psychological* denotes the body of knowledge (Greek -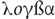, -logia) about psychical phenomena^3^.

Psychical phenomena (e.g., cognitions, emotions, and motivations) are conceived as located entirely internal and perceivable only by each individual itself and nobody else^4^ (Locke, 1999). Differences in temporal extension distinguish *experiencings* (Erleben), which are transient and bound to the here-and-now (e.g., thoughts, emotions), from *memorized psychical resultants* or commonly *experiences* (Erfahrung), which are, although accessible only through experiencings, temporally more extended in themselves (e.g., sensory and psychical representations, knowledge, abilities; with memorisation here broadly referring to any retention process). Unlike immaterial physical phenomena (e.g., heat, x-radiation), the psyche’s immaterial properties show neither spatial properties in themselves nor systematic relations to the spatial properties of physical phenomena to which they are bound (e.g., brain matter and physiology) and are therefore conceived as “non-physical”, reflecting complementary psyche-physicality relations (see Fahrenberg, 2013).

Internality, imperceptibility by others and lack of spatial properties differentiate psyche from possible externalizations in behaviors and language from which psychical phenomena can only be inferred indirectly. This has important implications for language-based methods like ratings as shown below.

##### Composite kinds of phenomena comprising both phenomena inseparable from the individual’s body and phenomena independent of it

In the TPS-Paradigm, three further kinds of phenomena are conceptually distinguished: *semiotic representations* (e.g., written and spoken language)—phenomena essential for rating methods—as well as *artificial outer-appearance modifications* (e.g., clothes) and *contexts* (e.g., situations) not considered here (see Uher, 2015a,c). They are conceived as *composites* because they comprise phenomena of different kind (as distinguished by the three metatheoretical properties) that are tightly interrelated with one another, forming a functional whole from which new properties emerge (Figure 2B). These new properties can be explored only by studying the composite’s constituents in their functional interdependence. Importantly, these composites are conceptual and not demarcated by physical boundaries (unlike, e.g., biological cells). Instead, their constituents are located apart from one another, which considerably complicates their exploration as semiotic representations illustrate.

**FIGURE 3:**
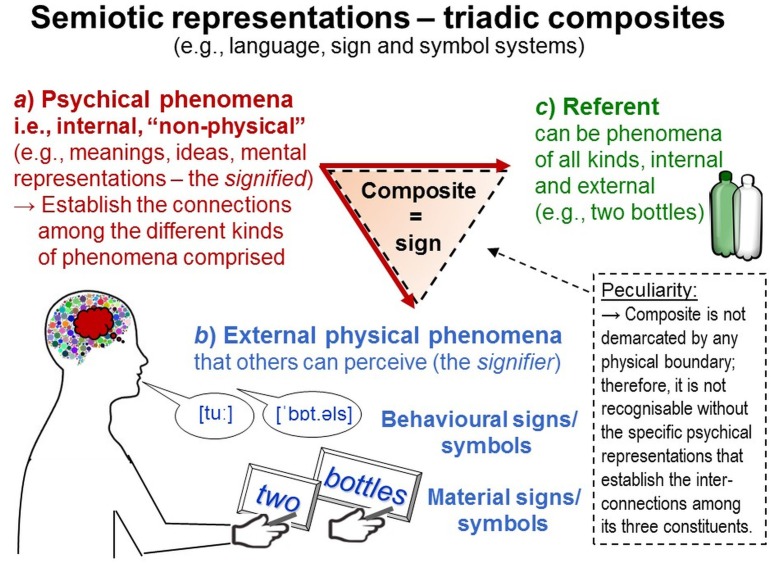
Semiotic representations: data. Semiotic representations are composites comprising both phenomena internal and phenomena external to individuals. Their intangible composite connections are established through the psychical constituent **(a)**, which enables a sign’s external physical constituent **(b)** to denote its referent **(c)** also in absence of the latter.

*Semiotic representations* (e.g., written language) are composites in which (a) particular *psychical constituents* (the *signified*; e.g., meanings, mental representations) are tightly interrelated with (b) particular *physical constituents external* to individuals’ bodies (the *signifier*; e.g., ink on paper, vocalizations) and (c) particular *referents* to which both refer and which may be located external or internal to individuals’ bodies. These three constituents form a functional composite from which new properties emerge—those of *signs* (sign includes the notion of *symbol* in the TPS-Paradigm). For example, a semiotic representation may comprise (a) ideas and meanings of bottles, (b) visual (graphic) patterns shaped like “BOTTLE” or “Flasche” (German for bottle), or acoustic (phonetic) patterns like [’bɒt.əl] or [‘flaʃə] and (c) some bottles to which both refer (Figure 3). Visual and acoustic patterns are external physical and can thus be perceived by others and used to decode the meanings and referents someone may have encoded in them. Importantly, meanings are not inherent to the physical signifiers in themselves but only *assigned* to them. Meaning construction occurs in people’s minds; it is internal and psychical (“non-physical”). The term semiotic *representation* highlights that individuals’ psychical representations are the essential component that interconnects a sign’s signifier with its referent. These three constituents are all located apart and not demarcated as an entity and therefore cannot be straightforwardly recognized as a composite. Socially shared as*sign*ments turn such composites into *signs*—but only for persons making such attributions (the signs’ psychical constituent). Such assignments are arbitrary and therefore vary (e.g., different alphabets; Vygotsky, 1962; Westen, 1996; Uher, 2015a). For these reasons, semiotic representations are complex and metatheoretically heterogeneous, involving external and internal, physical and “non-physical”, temporally extended and transient phenomena. This considerably complicates explorations, such as their function as data.

##### Excurse: data – semiotic representations used to encode information about the study phenomena

*Data* are signs (symbols) that scientists use to represent information about the study phenomena in physically persistent and easily perceivable ways. Thus, data are semiotic representations—composites comprising particular *physical constituents* (e.g., visible patterns like “two” or “2”) to which particular persons (e.g., scientists, observers) assign particular *meanings* (e.g., mathematical properties) and refer both to particular *referents*—the properties and phenomena under study (e.g., numbers of bottles; Figure 3).

Important types of data are *numerals* (apart from textual data). Numerals comprise physical constituents (e.g., visible patterns shaped like 1, 5, 10, and 50) to which individuals often—but not always—attribute the meaning of *numbers*. As such attributions are arbitrary, the meaning of numbers can also be attributed to other physical constituents (e.g., visible patterns shaped like I, V, X, L). Vice versa, different meanings can be assigned to the same signifiers that then constitute different signs (e.g., Roman numerals also represent alphabet characters). Consequently, *not all numerals represent numbers*. Whether or not numerals represent numbers depends on the meanings attributed by their creators—an important point for data generation.

Data, as they are signs (symbols), can be stored, manipulated, decomposed and recomposed, that is, *analyzed in lieu of the actual phenomena under study* (the referents) and in ways not applicable to these latter. But inferences about the study phenomena can be made *only if* the data represent relevant properties of these phenomena in appropriate ways. This is a further important point for data generation taken up again below.

### Methodological Framework

#### Data Generation Methods Are Determined by the Study Phenomena’s Modes of Perceptibility: Basic Principles and Method Classes

The three properties, because they describe modes of perceptibility under everyday conditions, also specify the ways to make phenomena accessible under research conditions. Therefore, these metatheoretical properties are used in the TPS-Paradigm to derive methodological principles and define basic method classes that cut across common classifications, which specify properties of data once these are generated (e.g., ‘qualitative’, ‘quantitative’; Uher, 2018a).

*External* phenomena (e.g., behaviors) are publicly accessible and can be studied without any mechanism standing between observer and observed using *observational methods*. *Internal physical* phenomena (e.g., brain), by contrast, are imperceptible under everyday conditions but can be made perceptible under research conditions using *invasive or technical methods* (e.g., surgery, X-ray).

*Temporally extended* phenomena (e.g., body morphology) do not change quickly, which facilitates perception and enables repeated perception of the same entity. *Transient phenomena* (e.g., behaviors, nerve potentials), by contrast, can be perceived and recorded only in the brief moments when they occur, thus real-time using so-called *nunc-ipsum*^5^ *methods* (e.g., observations, EEG).

*Physical* phenomena, both material and immaterial (e.g., morphology, heat), are spatially extended. Therefore, they can be captured with *physical methods*, which rely on the spatial extensions of materials that are systematically related to and more easily perceivable than the study phenomena (Uher, 2018a), such as mercury in glass tubes for measuring temperature (see Chang, 2004). *Psychical* phenomena, given their non-spatial (“non-physical”) properties, are inaccessible by any physical method and cannot be made perceivable by others. This unique property is used in the TPS-Paradigm to distinguish methods enabling access to psychical phenomena from those that cannot.

**FIGURE 4:**
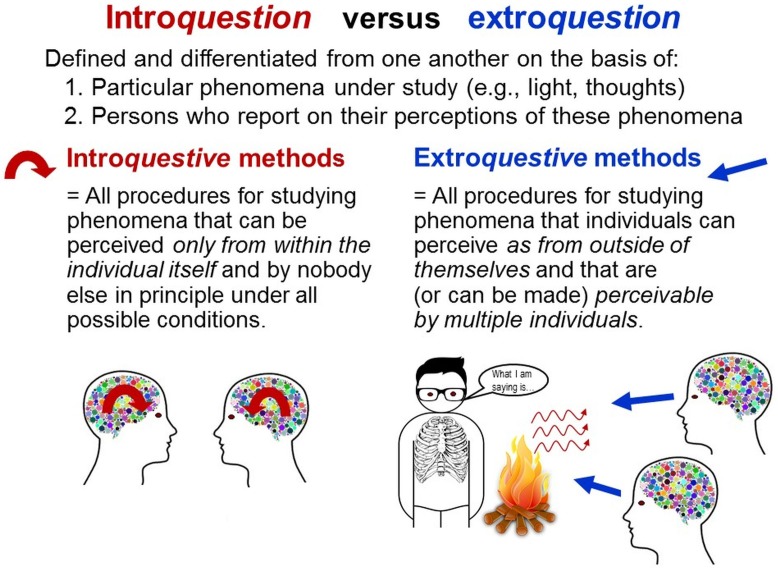
Introquestion versus extroquestion. Basic classes of methods for investigating psychical versus physical phenomena.

*Introquestive*^6^ *methods* are all procedures for studying phenomena that can be perceived *only from within the individual itself and by nobody else in principle under all possible conditions*. This applies to psychical phenomena, which can be explored by others *only indirectly* through individuals’ externalizations (e.g., behaviors, language). Accordingly, all methods of self-report and inner self-observation are introquestive. *Extroquestive*^7^ *methods*, by contrast, are all procedures for studying phenomena that *are or can (technically) be made perceptible by multiple individuals* (Figure 4). This applies to all physical phenomena (including internal and immaterial ones, e.g., inner organs, heat) because they can be made perceptible using invasive or technical methods (e.g., surgery, EEG). Joint perception of the same entity by multiple individuals (e.g., observers) is essential for data quality assurance (e.g., establishing intersubjectivity; see below; Uher, 2016a, 2018a).

Previous concepts of intro*spection* versus extro*spection* are distinguished from one another with regard to the studied individual by denoting its “inward perspective” versus “outward perspective”, respectively (Schwitzgebel, 2016). These two perspectives are, however, not perceived as separate channels of information. Instead, they are always merged in the multifaceted unity emerging from the composite of all perceptions available at any moment (Wundt, 1894). Therefore, introspection and extrospection cannot be differentiated as methods. By contrast, extro*question* and intro*question* are defined and differentiated on the basis of (a) the particular study phenomena (e.g., sounds, thoughts), considering that other internal and external phenomena can be simultaneously perceived, and of (b) the particular persons who perceive the study phenomena and generate from their perceptions data about these phenomena (Uher, 2016a).

These concepts highlight that psychophysical investigations of relations between sensory perceptions and physical stimuli (Fechner, 1860; Titchener, 1905)—commonly interpreted as introspective—are actually extroquestive methods. The physical stimuli (e.g., lights, sounds) are external to participants’ bodies and therefore perceivable also by the experimenters. Only because physical stimuli are extroquestively accessible can they be experimentally varied and compared with individuals’ subjective judgements. Thus, contrary to widespread assumptions, psychophysical findings about sensory perceptions cannot be generalized to perceptions of phenomena that are accessible only introquestively. Involvement of perceptions does not qualify investigations as introquestive because perceptions are always involved in any investigation (e.g., natural-science observation; Uher, 2016a, 2018a).

This perceptibility-based classification of data generation methods highlights that a phenomenon’s modes of accessibility determine *unequivocally* the class of methods required for its investigation. Each kind of phenomenon can be captured only with particular method classes and no method class allows for exploring all kinds of phenomena (see complementarity; Uher, 2018a). These are further important points for data generation taken up again below.

## Measurement and Quantification Across the Sciences

The TPS-Paradigm’s frameworks and the concepts outlined above are now applied to explore concepts of measurement and quantification, highlighting commonalities and differences among sciences.

### Measurement Versus Quantification

In psychology, quantification and measurement are often considered synonyms; but they are not the same. *Quantification* generally denotes the assignment of numbers, whereas *measurement* denotes a purposeful multi-step process, comprising operative structures for making such assignments in reliable and valid ways together with explanations of how this is achieved (Maul et al., 2018). Hence, not every quantification is an outcome of measurement (Abran et al., 2012).

### Concepts of Quantity and Early Measurement Theories

#### What Is a Quantity?

A *quantity* is a divisible property of entities of the same kind—thus, of the same quality. Two types are distinguished, multitudes and magnitudes (Hartmann, 1964).

*Multitudes* are discontinuous and discrete quantities that are divisible into indivisibles and discontinuous parts, which are countable—numerable—and therefore expressible as a number (e.g., persons, eyeblinks). Thus, multitudes are quantities by their ontological nature (Hartmann, 1964). *Magnitudes*, by contrast, are continuous and unified quantities that are divisible into divisibles and continuous parts. Magnitudes can be directly compared and rank-ordered in terms of ‘more’, ‘less’, or ‘equal’ (e.g., body length). By comparing a target property’s magnitude with the magnitudes of designated references of the same kind of property (e.g., length of units on rulers), which constitute multitudes and are thus countable, their ratios can be expressed as a measurement unit (e.g., meter) and a number (JCGM200:2012, 2012).

#### Early Measurement Theories and the Fundamental Problem of Psychological and Social-Science Measurement

From an epistemological analysis of counting and measuring (von Helmholtz, 1887), Hölder (1901) axiomatized equality/inequality, ordering and additivity relations among physical magnitudes, thereby laying the foundations for their measurement (Michell, 1997; Finkelstein, 2003). Quantities for which additive operations can be empirically constructed and quantities that can be derived from them led to further measurement concepts. In *fundamental (direct) measurement*, quantities are obtained directly (e.g., length). In *derived measurement*, the target quantity is obtained *indirectly* from relations between other directly measurable quantities (e.g., volume from length; Campbell, 1920). In *associative measurement*, the target quantity is obtained *indirectly* through measurement of another quantity with which it is systematically connected (e.g., temperature through length of mercury in glass tubes; Ellis, 1966; Chang, 2004).

Psychophysicists, pioneers of early psychology, studied equality and ordering relationships of sensory perceptions of physical stimuli (e.g., just-noticeable-differences and comparative judgements of light stimuli; Titchener, 1905), which is possible only because they constitute extroquestive explorations. But the properties of psychical phenomena in themselves, especially non-sensory ones (e.g., thoughts, emotions, and motivations), cannot be empirically added (concatenated) or derived from additive quantities. The possibility of their measurement was therefore rejected by the British Association’s for the Advancement of Science committee for quantitative methods (Ferguson et al., 1940; see also Kant, 1786/2016; Trendler, 2018). This led psychologists and social scientists to focus on relational models, operational theories and utility concepts (Michell, 1999; Finkelstein, 2003).

### Representational Theory of Measurement

**FIGURE 5:**
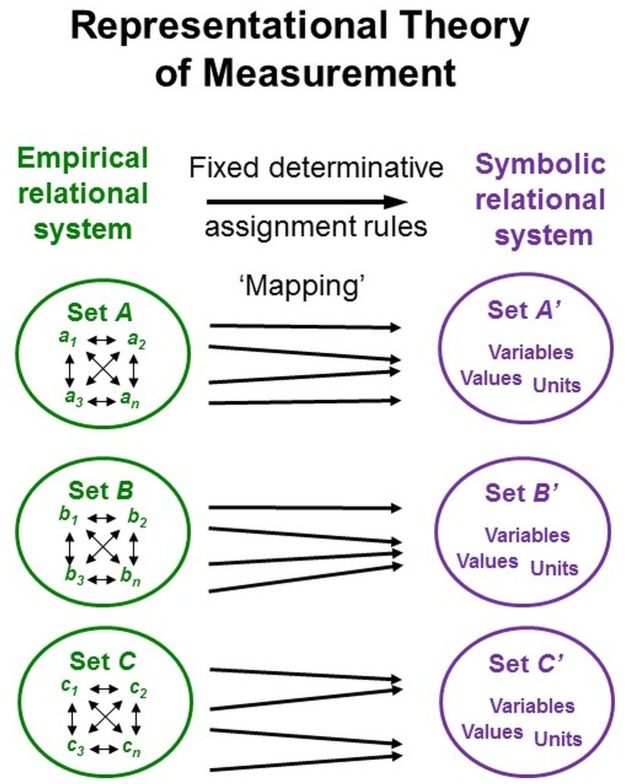
Representational theory of measurement. Key elements of representational systems frequently used in psychological and social-science concepts of measurement.

Representational theory of measurement, developed in the social sciences, formalizes (non-contradictory) axiomatic conditions by which empirical relational structures can be mapped onto symbolic relational structures, especially numerical ones (Krantz et al., 1971; Suppes, 2002). For measurement, these many-to-one mappings (homo- or isomorphisms) must be performed such that the study phenomena’s properties and their interrelations are appropriately represented by the properties and interrelations of the signs used as data (*representation theorem*). Permissible transformations specify how the numerical representations can be further transformed without breaking the mapping between the empirical relations under study and the numerical ones generated (*uniqueness theorem*; Figure 5; Vessonen, 2017).

In physical sciences and engineering, representational theory plays no role, however, despite its applicability (Finkelstein, 2003). This may be because it formalizes initial stages of measurement and important conditions of measurability but does not stipulate any particular measurement procedures (Mari et al., 2017). Another problem concerns establishing measurability (i.e., evidence of ordered additive structures of the same quality) because not just any mapping of numbers onto empirical relational structures constitutes measurement. But the appropriateness of particular numerical representations is often only assumed rather than established, thereby reducing the interpretability of the generated symbolic representation *regarding the empirical phenomena under study* (Blanton and Jaccard, 2006; Vessonen, 2017).

### Psychometric Theories of Measurement

Psychometric theories are concerned with statistical modeling approaches, building on various *positivist* epistemologies that focus on empirical evidence and predictive ability (*instrumentalist* focus) rather than on finding true explanations of reality. Therefore, some psychometricians apply *operationalist* epistemologies and determine study phenomena by the methods used for their exploration (Bridgman, 1927), such as by defining intelligence as “what an IQ-test measures” (Boring, 1923; van der Maas et al., 2014). This, however, reduces measurement to any number-yielding operation (Dingle, 1950). It also ignores that measurement results constitute information that can be understood also outside the specific context in which they were generated (Mari et al., 2017). The ability to represent information also in absence of their referents is a key feature of semiotic representations like data (Uher, 2015a, 2016b).

Psychometricians applying classical test theory or probabilistic latent trait theory (e.g., item response theory, Rasch modeling) sometimes build on *naïve realist* epistemologies by assuming ratios of invariant quantities exist in the world and independently of the methods used (Mari et al., 2017). Hence, they assume that ideal methods (e.g., purposefully designed rating scales) allow to empirically implement an identity function, turning pre-existing ‘real’ scores into estimated (manifest) scores—although with errors or only certain probabilities, which, however, can be defined with reference to the assumed ‘true’ scores or ‘latent trait’ scores, respectively. But this ignores that interactions between study properties and methods always influence the results obtained (Heisenberg, 1927; Bohr, 1937; Mari et al., 2017). In human-generated measurement, these interactions are intricate because they are mediated by the data-generating persons who perceive and interpret—thus interact with—both the study properties (whether located internally or externally) and the methods used (e.g., rating scales, observation schemes). Metrologists’ concepts of ‘humans as measuring instruments’ (Pendrill, 2014) and psychometrician’s concepts of rating scales as ‘measuring instruments’ do not reflect these triadic relationships.

### Metrological Concepts of Measurement and Scientific Quantification

**FIGURE 6:**
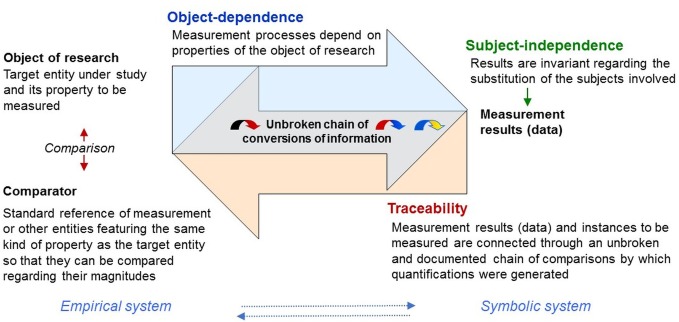
Traceability. Metrological concepts stipulate basic and testable elements of measurement procedures linking measurement results (data) with the phenomena and properties under study through traceable conversions of information. They provide key concepts by which symbolic (e.g., numerical) relational systems can be mapped onto empirical relational systems, which were left undefined in representational measurement theory.

To justify that quantifications can be attributed to the objects of research, metrologists define measurement as a purposive process comprising operative structures that establish evidence for its object-dependence (“objectivity”) and the subject-independence of its results (“intersubjectivity”; Figure 6 Frigerio et al., 2010; Mari et al., 2012, 2017). Importantly, in metrology, “*object*ivity” refers to the *object* of research and denotes that measurement processes depend on the objects and properties under study (therefore *object-dependence*)—compliant with complementarity. Results are “*intersubjective*” if they are “invariant with respect to the substitution of the involved subjects” (Mari et al., 2017)—thus, the persons generating and using them (therefore *subject-independence*). In psychology, by contrast, “objectivity” commonly denotes intersubjectivity in terms of independence from the investigator. It refers to the results not the process, thus confounding two metrological criteria of measurement.

An important way of establishing object-dependence and subject-independence is to implement *traceability*. Traceability requires measurement results to be systematically connected through an unbroken and documented chain of comparisons to a reference (comparator; Figure 6), which can be a measurement standard or the definition of a measurement unit through its practical realization (JCGM200:2012, 2012). This allows measurement results to be traced back to the particular instances of the properties measured (objects of research) and the particular comparisons and standards by which quantifications were obtained (empirical examples below).

These concepts stipulate basic and testable elements of measurement procedures by which ‘numbers can be mapped onto empirical relational structures’, thus allowing to establish evidence of measurability and intersubjectivity of the results obtained—key elements, left undefined in representational measurement theory (Figure 6). In the TPS-Paradigm, numerical data that fulfill these metrological criteria are called *scientific quantifications* as opposed to (subjective) quantifications in which these are not fulfilled.

## Person-Generated Measurement and Scientific Quantification: Basic Principles for Fulfilling Metrological Criteria in Psychology and Social Sciences

This section elaborates principles by which metrological concepts of measurement, although developed for physical phenomena, can also be met in investigations of “non-physical” phenomena, highlighting challenges and limitations.

### Establishing Object-Dependent Measurement Processes and Subject-Independent Results: Some Challenges

To connect objects of research (empirical relational structures) and measurement results (symbolic relational structures) through unbroken documented chains of comparisons, suitable operational processes must be established including explanations of how unbroken chaining is achieved (for issues of measurement uncertainty, not discussed here, see Giordani and Mari, 2012, 2014; Mari et al., 2017).

#### Constructs: Defining Theoretical Ideas

Psychological and social-science objects of research can be conceived very differently (e.g., behaviors, attitudes). Therefore, researchers must *theoretically define* the phenomena and properties of interest. Theoretical definitions describe the objects of research—in representative measurement theoretical terms, the empirical entities under study and their relational structures. Theoretical concepts are abstract and generalized ideas, which necessarily differ from their perceivable referents (Daston and Galison, 2007; Uher, 2015a). Abstract concepts (e.g., ‘personality’, ‘extraversion’, ‘social status’) describe complex constellations of phenomena that *cannot be directly perceived* at any moment but that are only theoretically *construct*ed as entities (therefore called *constructs*; Cronbach and Meehl, 1955). Hence, their theoretical definition is a matter of decision, which can but need not be intersubjectively agreed (see, e.g., different definitions and theories of ‘personality’).

#### Measurement Variables: Encoding Perceivable Qualities

As abstract ideas, constructs cannot be measured in themselves. Therefore, constructs are often called ‘latent’ in terms of ‘underlying’ and not directly perceivable, which often misleads people to reify constructs as real entities internal to individuals (e.g., ‘traits’ as psychophysical mechanisms; Uher, 2013).

To enable quantification, constructs must be *operationally defined*, thus, be related systematically to *specific indicators* that are directly measurable and used to quantify a construct indirectly. Erroneous analogies are sometimes drawn to indirect physical measurement, where the target quantity is derived from measurement of other directly measurable quantities (see above). But indirect measurement builds on natural connections among different kinds of quantities, which are experimentally identifiable whereas construct operationalization is a matter of decision, which may, but need not, be intersubjectively agreed (see, e.g., the different models and operationalizations of ‘personality’).

The complexity of constructs requires multiple indicators; but no set of indicators, however, large, can be all-inclusive (e.g., comprehensively operationalize ‘personality’). Constructs imply more meaning (*surplus meaning*) than the indicators by which they are operationalized. To ensure sufficient coverage, researchers specify a construct’s meanings in a theoretical framework of more specific sub-constructs and establish links to an empirical framework comprising sets of indicators. For example, popular models of the abstract construct of ‘personality’ comprise various more specific constructs (e.g., ‘extraversion’, ‘neuroticism’, ‘agreeableness’, and ‘conscientiousness’), each of which, in turn, comprises various sub-constructs (e.g., ‘gregariousness’, ‘assertiveness’) operationalized with various variables (e.g., rating items).

*Psychotechnical engineering*, where variables are purposefully chosen to operationalize theoretically defined constructs (following representational measurement theory), is aimed at generating aggregate scores for defined sets of variables (Cronbach and Meehl, 1955; Messick, 1995; Vautier et al., 2012). This differs from *psychometric engineering*, where construct definitions are derived from empirical interrelations among variables (following operationist assumptions; Thissen, 2001; Vautier et al., 2012). The Big Five personality constructs, for example, were derived from ratings on person-descriptors taken from the lexicon and are defined by these ratings’ empirical interrelations as studied with factor analysis (therefore, commonly called ‘personality’ factors; Uher, 2015d).

**FIGURE 7:**
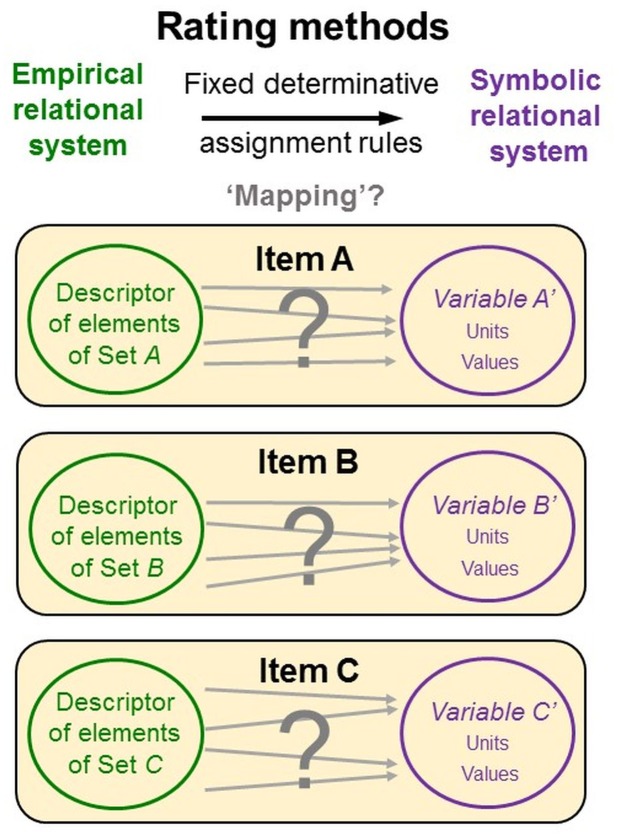
Rating items confound empirical and relational system. Rating scales serve both as descriptors of the empirical relational system and as elements of the symbolic relational system, thus confounding two key elements of representational measurement theory. Psychometricians provide neither definitions of each relational system nor specifications for assignments between them, leaving their interpretation and execution to raters’ intuitive judgments and decisions.

While these issues are well-known and intensely discussed, psychometricians hardly ever specify how the data-generating persons can actually identify the empirical relational system and execute the assignments to the symbolic relational system. This likely results from the deficiencies of representational measurement theory and psychometric theories but also from the “non-physical” objects of research and language-based data generation methods. Specifically, constructs are abstract ideas that ‘exist’ as entities only in people’s mind and language. When concepts constituted by words are explored with methods constituted by words, it is difficult to distinguish the methods from the measures of the object of research (Lahlou, 1998; Uher, 2015d). Rating scales serve both as descriptors of the empirical relational system and as elements of the symbolic relational systems, thus *confounding two key elements of representational measurement theory*. Psychometricians provide raters neither with clear definitions of each relational system nor with specifications of the assignments to be made between them, leaving their interpretation and execution to raters’ intuitive judgments and decisions (Figure 7).

**FIGURE 8:**
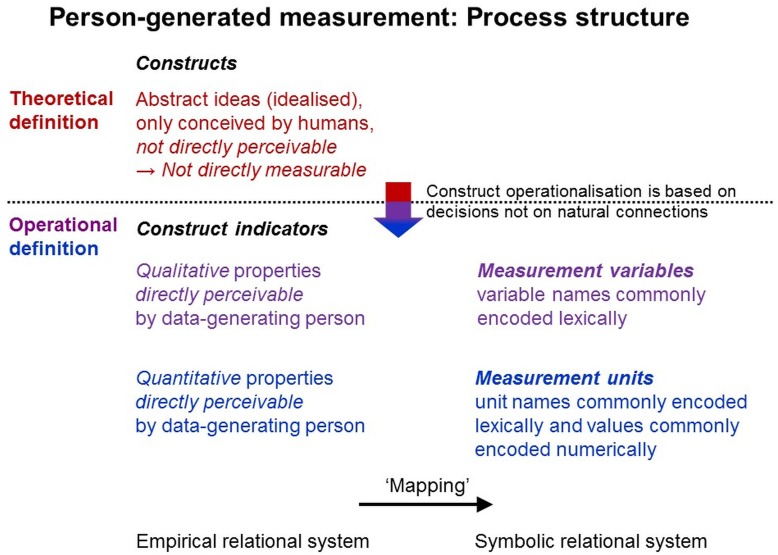
Theoretical definition and empirical operationalization. Process structure for measurement directly by persons in research on individuals.

As data generation requires interaction with the objects of research, persons must be able to *directly perceive* them. Consequently, data generation methods must be used that match the study phenomena’s modes of perceptibility (see above). Researchers must define the study phenomena in terms of their *perceivable qualitative properties* and must specify the variables in which they are (commonly lexically) encoded (Figure 8).

#### Measurement Units: Encoding Perceivable Quantities

For each measurement variable, researchers must then define *measurement units*. As they belong to the *same* variable, units refer to properties conceived as identical or at least sufficiently similar—thus, of the *same quality*.

Different types of units are used. Nominal units encode either more specific qualities or, as binary units, absence versus presence of the quality of interest, whereas rational, interval and ordinal units encode divisible properties of the quality studied, thus quantitative properties. For each unit type, permissible transformations are specified that maintain the mapping to the empirical relational system under study (Stevens, 1946). Hence, the empirical relational system’s properties determine which unit type can be used. For person-generated quantification, researchers must define divisible properties of the study phenomena that are or can be made *directly perceivable* during data generation (Figure 8).

#### Encoding Procedure: Defining Fixed and Unchanging Assignment Rules

For measurement, the *same* properties must *always* be encoded with the *same* signs so that the data obtained always represent the *same* information and can be understood and used by others in the *same* way, thus subject-independently. This presupposes explicit assignment rules (e.g., many-to-one mappings), variables, units and values that are *fixed and unchanging*. Metatheoretically speaking, the symbolic systems (e.g., observational encoding scheme) must be intersubjectively understood with regard to the referents and meanings they are meant to semiotically encode.

### Decisions to Be Made by the Person Generating the Data During Measurement Execution

**FIGURE 9:**
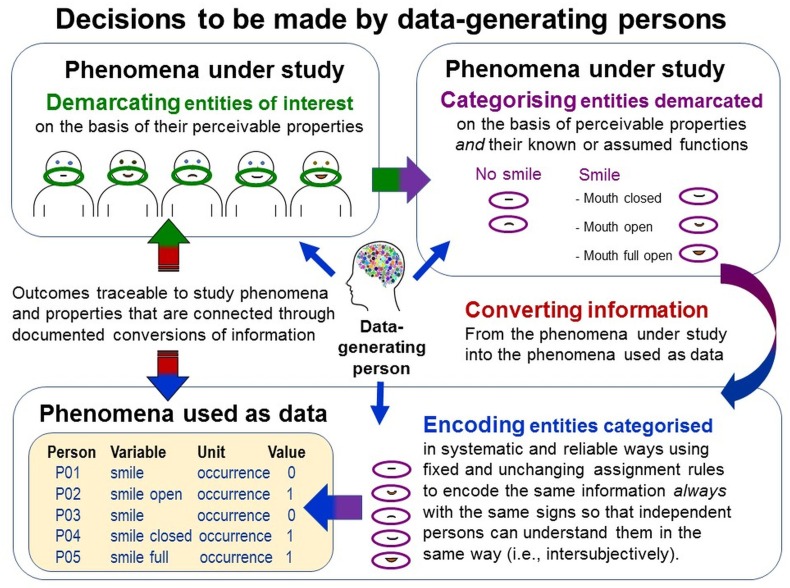
Decisions to be made by data-generating persons. Data generation requires complex decisions about demarcating, categorizing and encoding the entities of interest. They are particularly challenging when study phenomena feature variable perceivable properties (e.g., variable spatio-temporal extensions of behaviors). For example, smiles vary in spatial extensions; humans can turn their mouth corners up in various ways, with mouth open or closed, with or without laughter lines around their eyes, and all this at various levels of intensity. Given this, what entity can be demarcated and categorized as one (*n* = 1) smile? Smiles also vary in temporal extension. Are quick and long-lasting smiles events of the same kind? When does one smile end and another one start? Thus, which particular demarcable entities can be considered to be sufficiently similar to categorize them as being of the same kind? Making these decisions explicit is important for establishing traceability of the generated data.

To execute a measurement task, persons must have certain abilities and make various decisions (Figure 9), which form inherent parts of *data generation methods* (Uher, 2018a). Extroquestive accessibility of study phenomena enables multiple persons to jointly perceive the same entity. This facilitates establishing intersubjective consensus in making these decisions (i.e., subject-independence).

#### Demarcating the Entities of Interest Using Perceivable Properties

First, in the multitude of a study phenomenon’s perceivable properties, data-generating persons must be able to demarcate the entities of interest in reliable and systematic ways. They must decide which pieces of information should be demarcated in what ways using perceivable similarities and dissimilarities. Variations in perceivable properties (e.g., in spatio-temporal extensions in behaviors) complicate these decisions (e.g., which demarcable entities are sufficiently similar to count as being of the same kind; Figure 9).

#### Categorizing Demarcated Entities Using Theoretical Considerations

Then, data-generating persons must decide how to categorize the demarcated entities using perceivable properties but also similarities and differences in their *known or assumed functions and meanings*—thus, theoretical and contextual considerations. For example, the behavioral acts of slapping someone to kill a mosquito and to bully that individual feature almost identical perceivable properties but differ in function and meaning; whereas smiling, talking and shaking hands have similar social functions but differ in their perceivable spatio-temporal forms (Figure 9). This shows why data generation is always theory-laden; as Einstein already said “it is the theory which decides what can be observed” (Heisenberg, 1989, p. 10). When researchers provide no system for categorizing the entities under study, as in many so-called ‘data-driven’ approaches, then the data-generating persons must use their own implicit theories to accomplish this task.

#### Converting Information About Categorized Entities Into Semiotically Encoded Information

Thereafter, data-generating persons must represent perceived occurrences of the thus-categorized entities into the signs used as data. When information from one kind of phenomenon is represented in another one, this is called *conversion* in the TPS-Paradigm (Uher, 2018a). For systematic and standardized conversions of information from the empirical into the symbolic relational system, scientists must specify which pieces of information from the study phenomena should be demarcated, categorized and semiotically encoded in what ways. That is, scientists must define the three constituents of the signs used as data (see Figure 3) such that the data-generating persons can execute the measurement operations.

#### Scientific Quantifications Generated Directly by Persons: General Preconditions and Challenges

Psychometricians are rather unconcerned with all these decisions raters have to make during data generation. Instead, psychometricians apply sophisticated methods of data modeling (e.g., Rasch modeling) to demonstrate that the data—once raters have produced them—exhibit quantitative structures. But data analysis cannot *add* fundamental properties that have not been encoded in the raw data. To what extent are persons actually able to *directly generate* scientific quantifications (i.e., quantitative data that are object-dependent and subject-independent) *during data generation*?

**FIGURE 10:**
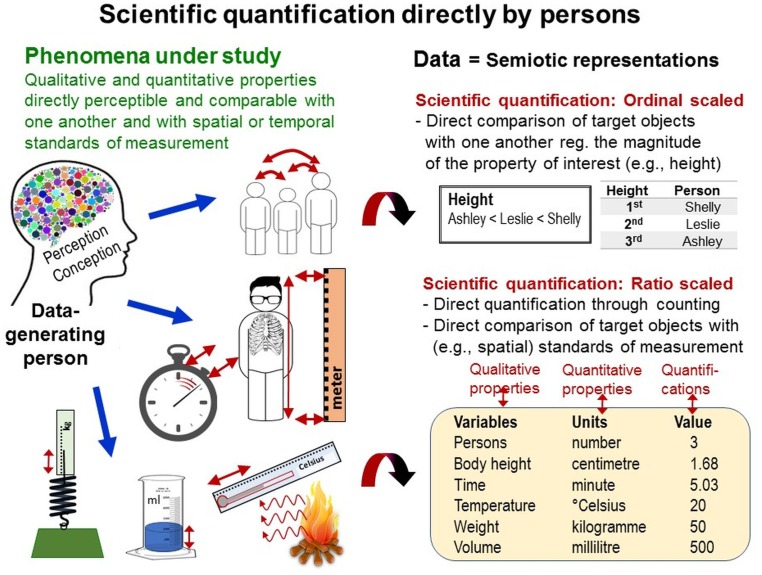
Scientific quantification directly by persons. Scientific quantification directly by persons during data generation is possible only by counting multitudes and through direct perceptual comparison of the magnitudes of the phenomena and properties under study with one another and with the magnitudes of spatial and temporal standards of measurement.

For interval and ratio-scaled direct quantifications, spatial standard units of measurement are widely used (e.g., yard sticks for length measurement). Distinct entities (i.e., multitudes; e.g., rope jumps) can be directly counted. If not applicable, persons can compare several entities with one another—provided these can be perceived in close spatial *and* temporal proximity together—to determine their relative magnitude regarding the quality of interest (e.g., body height, intensity), thus enabling ordinal-scaled quantifications^8^ (e.g., highest, second highest, third highest; Figure 10).

But persons’ abilities to count or directly compare the entities of interest with one another or with spatial standards of measurement are often compromised in momentary and highly fluctuating phenomena featuring variable properties. For example, the dynamics of behaviors often hinder applications of spatial standards of measurement (e.g., to quantify movements). Direct comparisons between behavioral acts are complicated both within individuals because previous acts have already ceased to be and between individuals because individuals seldom behave spatio-temporally in parallel with one another (as arranged in races). To solve this problem, behavioral scientists (e.g., biologists) apply observational methods enabling time-based measurement, whereas psychologists and social scientists primarily use rating methods. These two methods are now explored in detail and compared with one another.

## Quantitative Data Generation With Rating Methods Versus Observational Methods: Possibilities and Limitations for Fulfilling Metrological Criteria

The TPS-Paradigm’s frameworks and the metrological criteria of scientific quantification are now applied to deconstruct the demands that different methods of quantification impose on data-generating persons, contrasting rating methods with behavioral observations (starting with the latter). These elaborations are illustrated by the example of individual-specific behaviors as study phenomena (behavioral parts of ‘personality’). To be specific to individuals, behavioral patterns must vary among individuals and these differences must be stable over some time (Uher, 2013, 2018b). But neither differential nor temporal patterns can be directly perceived at any moment. As behaviors are transient, fluctuating and dynamic, individual behavior patterns cannot be straightforwardly measured either (Uher, 2011). This considerably complicates quantifications of individual-specific behaviors.

### Demands Placed on Observers

#### Targeted Perception and Real-Time Demarcation, Categorization and Encoding

Observation, unlike looking or watching, involves targeted and *systematic perception* of the phenomena and properties under study. As behaviors are transient, observers must target their perceptions to relevant properties and must demarcate and categorize behavioral events *in the brief* moments while they occur, thus real-time using nunc-ipsum methods (see above). To achieve this in standardized ways *while observing* the continuous flow of dynamically changing events, observers must know by heart all elements of the empirical relational system under study (e.g., all definitions of behavioral acts specified in the ethogramme), all assignment rules and all variables, units and values that constitute the symbolic relational system, thus the data.

To meet these demands, observers are instructed and trained. Training is possible because behaviors are extroquestively accessible, which facilitates intersubjective perception and discussion about the decisions required to generate data. Observers’ performances are studied as agreement between codings generated by independent persons observing the *same* behaviors in the *same* individuals and situations at the *same* occasions. This subject-independence is statistically analyzed as inter-observer (inter-coder) reliability. Behaviors’ extroquestive accessibility also facilitates the design of object-dependent observation processes involving unbroken documented chains of comparisons (see Figure 6). Video-based coding software allows observers to mark the video sequences in which particular behaviors occur so that their demarcation, categorisation and encoding can be traced to the specific behaviors observed (as recorded on video) and to the ways they were quantified.

#### Defining the Empirical Relational System: Specifying All Studied Elements of the Sets B, S, I, and T

**FIGURE 11:**
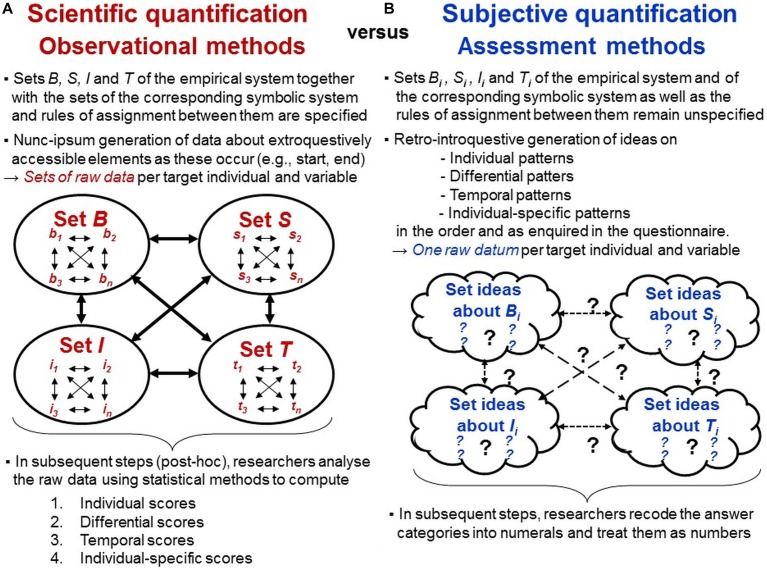
Scientific versus subjective quantification. Processes involved in the generation of quantitative data using **(A)** observational methods versus **(B)** assessment methods by the example of investigations of individual-specific behaviors (habitual behaviors forming part of an individual’s ‘personality’).

To enable observers to perceive, demarcate, categorize and encode behaviors in systematic and standardized ways, researchers must specify all phenomena to be quantified (i.e., all elements of the empirical relational system) in terms of their perceivable qualitative and quantitative properties. For investigations of individual-specific behaviors, this involves the set *B* of all behaviors studied and the set *S* of all situations in which they are observed (considering the context-dependent meanings of behaviors; Uher, 2016b). Researchers must also specify the set *I* of individuals studied as well as the set *T* of occasions and periods of time in which their behaviors are recorded (Figure 11A).

The sets of individuals (e.g., sample characteristics) and times studied (e.g., observation time per individual) are specified in every method section. Situations can be defined on more abstract levels as nominal situations (e.g., location, test condition) or on more fine-grained levels such as regarding specific interpersonal situations (e.g., being approached by others). This requires observers to demarcate and categorize situational properties *in addition to* the behavioral properties studied, thus further increasing the demands placed on them. For such fine-grained analyses, researchers often use video-based techniques enabling deceleration of the flow of events and repeated observations of the same instances.

The perceivable qualities of behaviors can often be interpreted differently regarding their possible functions and meanings. To enable categorisation, researchers must specify the theoretical interrelations of the behaviors studied as well as their possible contexts and observers must know these by heart. Observational designs must be developed that are practically feasible given the used settings (e.g., restricted or unrestricted), sampling methods (e.g., behavior or time sampling) and recording techniques (e.g., manual or computerized recording). Defining the studied elements’ theoretical interrelations within and between the empirical sets *B, S, I*, and *T* studied is prerequisite for specifying the corresponding symbolic relational system (therefore indicated with primes) involving the sets *B*′, *S*′, *I*′, and *T*′ as well as the rules for assignments between both (Figures 4, 11A).

#### Measuring and Quantifying Behavioral Events Real-Time – Possibilities and Limitations

Transience and pronounced spatio-temporal variations of behaviors require that observers decide flexibly about how to demarcate events, thus often precluding comparisons with *spatial standards of measurement*. Therefore, behavioral events—with all perceivable spatial variations as defined by the researchers—are often encoded only in their occurrence or non-occurrence using binary units (see Figure 9). Scientific quantifications are then generated through comparison with *temporal standards of measurement*. Such quantifications, as they are based on behaviors’ temporal properties, may differ from quantifications that could be obtained from their spatial properties. Time-based measurement puts high demands on observers because they must monitor time in addition to the behavioral and situational properties studied. It also requires clear specification of the perceivable divisible properties used for quantification (see Figure 9). Software- and video-based observation technologies facilitate the nunc-ipsum recording of occurrences, onsets and ends of binarily encoded behavioral events, producing time-based log-files (Uher, 2013, 2015b; Uher et al., 2013a).

Summarizing, behavioral observations place high demands on the data-generating persons. They show that persons’ abilities to directly generate quantifications that meet axioms of quantity and measurement are very limited. This often confines observational data to nominal formats; but these data are clearly defined and traceable and thus suited to generate scientific quantifications *post hoc* (see next). By recording the events of interest in nominal units while or immediately after they occur (nunc-ipsum), observers have already completed their task. They are required neither to memorize events observed, nor to directly quantify them nor to mentally compute their empirical occurrences or interrelations within and across the empirical sets *B, S, I*, and *T*. Such computations are made by researchers in subsequent steps of data *analysis* using the elements of the symbolic relational system generated.

#### After Observations Are Completed: *Post hoc* Generation of Ratio-Scaled Data From Nominal-Scaled Raw Data

Nominal data indicate classification, which is essential for measurement but not yet quantification. Because nominal units disjunctively encode occurrence or non-occurrence of qualitative properties, nominal-scaled raw data can be used to generate ratio-scaled quantifications, which meet the axioms of quantity and quantification, *post hoc—after raw data generation has been completed*. Generating ratio-scaled quantifications of individual-specific behaviors (‘personality’ scores) requires three steps (Figure 11A; Uher, 2011, 2013).

First, to generate data reflecting *individual patterns*, each individual’s raw data are aggregated over specified time periods, thus, they are *temporally standardized*. Formally speaking, in the symbolic relational system, the nominal-scaled data (multitudes) of each studied element *b*′_n_ for each studied element *i*′_n_ within each studied element *s*′_n_ are aggregated (counted) and then related to all studied elements *t*′_n_ of the set *T*′. Because non-occurrence of events defines an absolute zero point, the data thus-generated are *ratio-scaled*. Most behavioral coding software executes this step automatically (e.g., computing durations and frequencies).

Second, to generate data reflecting patterns of *individual differences*, individuals’ data must be *differentially standardized* within the sample and each situation studied (e.g., using *z*-standardization). Formally speaking, the data generated in step 1 for each element *i*′_n_ within each element *b*′_n_ and each element *s*′_n_ are statistically standardized across the entire set *I*′ of individuals (Figure 11A). *Differential standardization* transforms data reflecting absolute quantifications into data reflecting *relative* between-individual differences. As these transformations are made explicitly and *post hoc*, individuals’ absolute scores can always be traced for interpretation and possible re-analyses as well as for comparisons with other sets of individuals, thus fulfilling the criterion of object-dependence of measurement outcomes. Differential standardization enables direct comparison of individuals’ relative scores among behavioral variables of different kind (e.g., frequencies, durations) both within and between individuals. It also enables statistical aggregation into more abstract and composite variables on the basis of algorithms (e.g., different weighting of behaviors) that are specified in the theoretical definitions of the empirical relational system. Importantly, these comparisons and aggregations are always made with regard to the differential patterns reflected in the data, not with regard to individuals’ absolute scores (computed in step 1) because these may generally vary across behaviors and situations (Uher, 2011).

Third, differential patterns can reflect *individual-specificity* only if they are stable across time periods longer than those in which they were first ascertained and in ways considered to be meaningful (e.g., defined by test-retest correlation strength; Uher, 2018b). Hence, identifying individual-specificity in behavior requires *temporal analyses* of differential patterns that are defined by certain temporal patterns in themselves (Uher et al., 2013a). Therefore, the symbolic set *T*′ of occasions and spans of time is divided into subsets (e.g., *t*′_1_ and *t*′_2_), and steps 1 and 2 are performed separately on these subsets to enable between-subset comparisons for test–retest reliability analysis. Sufficient test–retest reliability provided, the differential patterns obtained in step 2 are then aggregated across the subsets *t*′_n_ to obtain data reflecting *ratio-scaled quantifications* of *individual-specificity* in behaviors (Figure 11A).

Importantly, this *post hoc* data processing is done by researchers and constitutes first steps of data analysis, which is independent of observers’ data generation task. These analytical steps are described here to highlight the complexity of the comparisons required to scientifically quantify individual-specific behaviors. This puts into perspective the demands placed on raters.

### Demands Placed on Raters

#### Quantifying Individual-Specific Behaviors Directly – An Impossible Requirement

To quantify individual-specific behaviors with rating methods, relevant behaviors are described in sets of statements, called *items*, that constitute a rating ‘instrument’ (e.g., questionnaire, inventory, and survey). Persons, the raters, are asked to judge the behaviors described (e.g., “tends to be lazy”) regarding, for example, their occurrences, intensity or typicality for a target individual. Raters are asked to indicate their judgements on rating scales comprising a fixed set of *answer categories* often labeled lexically (e.g., “agree” or “neither agree nor disagree” and “disagree”). Hence, raters are asked to *directly quantify* individual-specific behaviors.

But in everyday life and without recording technologies, persons often cannot *directly* quantify even single behavioral events (see section “Demands Placed on Observers”). Quantifying individual specificity in behaviors (or other kinds of phenomena) requires quantifying not only single events and individual patterns in many behaviors but also differences among individuals and over time. But in transient, dynamic and fluctuating phenomena, differential and temporal patterns cannot be directly perceived and thus cannot be quantified at any moment. Individual specificity is not an entity one could directly perceive but an abstract idea constructed by humans. For this construction, human language is essential.

#### Language – Essential for Abstract Thinking but Also Misleading

Language allows persons to semiotically represent perceivable phenomena (e.g., concrete behavioral acts) in single words (e.g., “shout”, “kick”). This allows perceivable qualities to be made independent of their immediate perception and to abstract them into objects, thus *reifying* them (“aggression”). This so-called *hypostatic abstraction* (Peirce, 1958, CP 4.227) enables people to develop not only concrete words that refer to directly perceivable phenomena but also abstract words that refer to ideas and concepts describing phenomena that are distant from immediate perception (Vygotsky, 1962) or complex and imperceptible in themselves—such as constructs of ‘personality’ (e.g., “aggressive*ness*”). Signs (e.g., rating items) therefore cannot reflect the referents they denote (Figure 3) in the same ways as individuals can perceive them.

People (including scientists) often tend to mistake linguistic abstractions for concrete realities. This so-called *fallacy of misplaced concreteness* (Whitehead, 1929) misleads people to assume that the complex phenomena described with abstract terms (e.g., in rating items) could be directly perceived. It also occurs when people encode their ideas about individual specificity in abstract terms (e.g., ‘personality’, ‘traits’, ‘character’, or ‘dispositions’), and then treat these abstractions as real entities that they assume to underlie individuals’ feeling, thinking and behaving and thus to be located internally. This entails explanatory circularity (Uher, 2013, 2018b).

Further challenges occur because semiotic representations contain implicit structures in both their external physical constituents (e.g., phonetics) and the particular meanings of the referents assigned to them (e.g., semantics). These implicit structures and meanings are not readily apparent because no physical border demarcates a sign’s three constituents as an entity (see above). This entails intricacies for language-based methods like ratings.

#### Standardized Rating Items Do Not Reflect Standardized Meanings—Instead, Their Meanings Vary Within and Between Individuals

Like in observations, variables and units of rating scales (as elements of the symbolic relational system) are predetermined and fixed. But unlike in observations, raters are commonly neither instructed nor trained to interpret and use them in standardized ways—thus in how to understand the given symbolic relational system and to relate it to the empirical relational system under study (in fact, both systems are confounded in rating methods; see Figure 7).

Rating scales are worded in everyday language in abstract and generalized ways to make them applicable to diverse phenomena, events and contexts without specifying any particular ones. Therefore, raters must use their common-sense knowledge to interpret and contextualize the given scale and to construct specific meanings for the rating task at hand. Common-sense categories are, however, not as well-elaborated and disjunctive as scientific categories but often fuzzy and context-sensitive, enabling flexible demarcations (Hammersley, 2013). To reduce cognitive effort, raters may interpret items on the abstract level on which they are worded—the semantic level (Shweder and D’Andrade, 1980; Block, 2010). Semantic processing may be triggered especially by highly inferential items requiring judgments of the social valence, appropriateness, and normativity of individual behaviors (e.g., “respectful”, “socially adapted”) or of their underlying aims and motivations (e.g., “helping”).

As meanings are not inherent but only as*sign*ed to the physical constituents of signs (e.g., phonemes, graphemes), meanings vary. For popular personality questionnaires, substantial within- and between-individual variations in item interpretations have meanwhile been demonstrated, highlighting that—contrary to common assumptions—standardized items represent not standardized meanings but broad and heterogeneous *fields of meaning* (Valsiner et al., 2005; Rosenbaum and Valsiner, 2011; Arro, 2013; Lundmann and Villadsen, 2016; Uher and Visalberghi, 2016).

In personality psychology, broadly worded rating items are known to be related to broader ranges of more heterogeneous and less specific behaviors and situations, thus representing more diverse aspects of given constructs (Borkenau and Müller, 1991). So far, such fidelity-bandwidth trade-offs were not regarded problematic because more abstract items have higher predictive validity for broader ranges of behaviors (e.g., job performance; Ones and Viswesvaran, 1996). Following instrumentalist epistemologies, broad rating items were even considered necessary to match the breadth of the criteria to be predicted (Hogan and Roberts, 1996).

**FIGURE 12:**
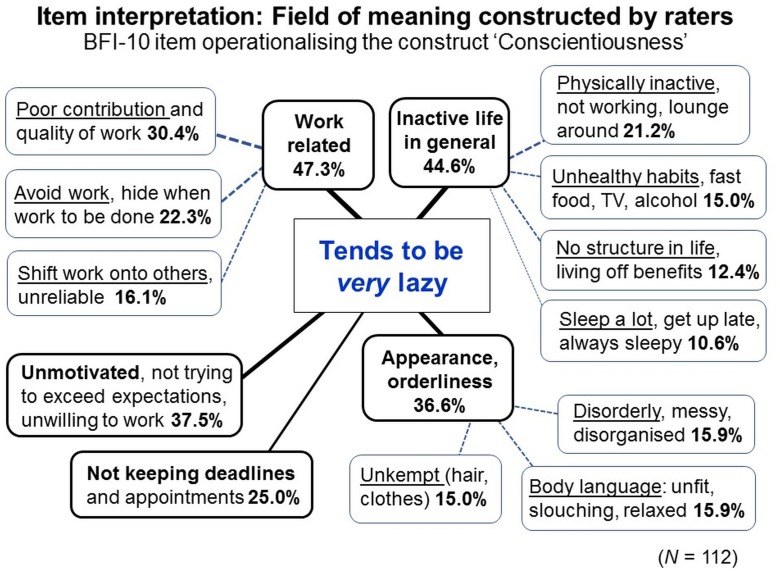
Diverse item interpretations spanning a field of meaning. Interpretations provided by *N* = 112 raters for a fictitious target person scoring high on the item. Main-themes (bold frames) summarize sub-themes of similar interpretations (non-bold frames). Numbers indicate percentages of raters who provided given interpretations; for the main-themes, each rater was counted just once even when they provided multiple similar interpretations as specified in the corresponding sub-themes.

From a measurement perspective, however, fidelity-bandwidth trade-offs are highly problematic because they entail lack of traceability of what has actually been encoded in the data. An example illustrates this. Interpretations of “tends to be lazy”, operationalizing the construct Conscientiousness in a popular personality inventory (BFI-10; Rammstedt and John, 2007), varied considerably within and among 112 raters. Raters variously associated this item with different behaviors and situations related to work, an inactive life style, appearance and orderliness, not keeping deadlines and lack of motivation (Figure 12; Uher and Dharyial, unpublished). This diversity may reflect the well-known fidelity-bandwidth trade-offs of broadly worded items. But importantly, every rater provided on average only two different interpretations (*M* = 2.08; *SD* = 0.92; range = 1–5). Thus, the single raters did not consider the item’s broad field of meaning that it may generally have in their socio-linguistic community. Instead, when judging the target person, different raters thought of very different behaviors and contexts; some considered “sleeping a lot”, others “shifting work on others”, still others “eating fast food”, or “not keeping deadlines” (Figure 12). This may explain the substantial variations in internal consistencies of personality scales across countries (see above).

Moreover, raters’ item interpretations can also go beyond the bandwidth of meanings that researchers may consider. A study involving five-method comparisons showed that, despite expert-based item generation, raters’ item interpretations clearly referred also to constructs other than those intended to be operationalized; raters’ and researchers’ interpretations overlapped to only 54.1–70.4% (Uher and Visalberghi, 2016).

Variations in item interpretation are an unavoidable consequence of the abstract and generalized wording of items and the necessity for raters to apply them to specific cases, and therefore occur despite careful iterative item selection (Uher and Visalberghi, 2016). They show that, for different raters, the same item variables do not represent the same meanings (symbolic relational system); consequently, raters do not have the same empirical relational system in mind. This precludes subject-independence and also limits possibilities to establish object-dependence. These issues are ethically problematic because ‘personality’ ratings are used not only for making predictions (following instrumental epistemologies) but also to identify properties that are attributable to the target individuals (following naïve-realist epistemologies).

#### Unknown Demarcation, Categorization and Encoding Decisions: The Referents Raters Consider for Their Ratings Remain Unspecified

A key feature of rating methods is the *introquestive data generation in retrospect*. This allows persons to generate data any time, in any situation (e.g., online), and even in complete absence of the phenomena (e.g., behaviors) and individuals under study. This contributes to the enormous efficiency of ratings (Uher, 2015e)—but has numerous methodical implications.

Persons can encode in data relevant information about the study phenomena *only if* they can directly perceive the phenomena and properties under study during data generation. Direct perceptibility is prerequisite for establishing object-related measurement processes (see above). But quantitative ratings inherently involve also comparisons among individuals or over time or both. To rate (one’s own or others’) individual-specific behaviors, raters must consider past behaviors and situations, thus phenomena no longer extroquestively accessible. Raters can form such judgments only by retrieving pertinent information from memory; therefore, ratings *are long-term memory-based introquestive methods*^9^ (Uher, 2018a).

Human abilities to reconstruct memorized events are generally constrained, susceptible to various fallacies and influenced by situational contexts (Shweder and D’Andrade, 1980; Schacter, 1999; Schacter and Addis, 2007). Therefore, assessments are influenced by raters’ motivations and goals (Biesanz and Human, 2010). Moreover, in individuals’ psychical systems, past events are stored not in the forms as once perceived but only in abstracted, integrated and often lexically encoded form (Le Poidevin, 2011; Valsiner, 2012). Individuals can base their ratings only on the outcomes of their past processing of past perceptions and conceptions; thus, on the beliefs, narratives and knowledge they have developed about individuals in general and the target individual in particular. Ratings cannot encode (habitual) behaviors in themselves, as sometimes assumed, but only the psychical and semiotic representations raters have developed about them—which are phenomena very different from behaviors (see above; Uher, 2013, 2015d, 2016a,b).

When raters’ responses are restricted to ticking boxes on standardized scales, not only remain differences in item interpretations unknown but also raters’ decisions on how to demarcate, categorize and encode the phenomena and properties that *they* consider as the referents of their ratings (elements of the empirical relational system). Formally stated, the elements of the set *B*_i_ of ideas about behaviors, the set *S*_i_ of ideas about behavioral situations, the sets *I*_i_ of ideas about individuals, the set *T*_i_ of ideas about occasions and spans of time as well as the ideas about these elements’ empirical occurrences and interrelations that raters implicitly consider cannot be specified (Figure 11B). Consequently, raters’ decisions during data generation and their degree of standardization and reliability cannot be analyzed. With inter-rater reliability, psychometricians analyze only agreement in the data sets produced (symbolic relational system) but not agreement in the ways in which raters demarcate and categorize information from the study phenomena (empirical relational system) and convert and encode them in the data (representational mapping between both systems). Insufficient or even lacking specification of the objects of research (Figure 7) hinders the design of object-dependent measurement processes and compromises the interpretability of data and findings.

In rating methods, every item variable is commonly used only once to generate one single datum per target individual and rater. In extroquestive nunc-ipsum methods like observations, by contrast, measurement variables can be used without limitation to encode defined elements of the empirical relational system as often as these may empirically occur—observational methods are matched to the study phenomena (object-dependence). The same variable can be used to generate entire data sets per target individual and observer (Figures 11A,B). In rating methods, by contrast, variables are presented in a fixed order predetermined by the researcher and that is mostly random with regard to the empirical relational systems they are meant to operationalize. Consequently, occurrences of events in raters’ minds are triggered by and adapted to the given rating scale—*the study phenomena are matched to the methods rather than vice versa* (Westen, 1996; Toomela and Valsiner, 2010; Omi, 2012; Uher, 2015d,e).

#### Unknown and Changing Units and Assignment Rules

Scale categories are commonly labeled with global terms (e.g., “strongly”, “often”), icons (e.g. 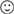, 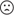), numerals or segmented lines. Given the well-known fidelity-bandwidth trade-offs of broadly worded rating items, researchers commonly assume that, for any given rating, raters consider a broader range of evidence to form an overall judgment that they then indicate in a single score on the scale. But how do raters actually choose the answer box on the scale? How do they interpret and use quantitative scale categories at all?

To constitute measurement units, scale categories must refer to the same quality as defined by the item variable. The different scale categories assigned to each variable must represent divisible properties, thus different quantities of that quality. These categories’ interrelations must adequately reflect the interrelations among the quantities of the empirical relational system that they encode. Thus, a lower score on the scale must indicate a lower quantity of the quality under study than a higher score on that same scale. Statistical aggregation across different items—a common practice in psychometrics—presupposes that the scale units have the same meaning for *all* the item variables for which they are used; similarly, metric units of weight (e.g., kg, lb) have the same meaning for all kinds of objects, whether stones, persons or feathers. Statistical aggregation across different raters—another common practice in psychometrics—presupposes that the different raters interpret and use the same scale in the same way. Similarly, different weighing machines, no matter how constructed, should be standardized (i.e., calibrated) and provide the same results for the same object. Hence, measurement outcomes should be subject-independent.

**FIGURE 13:**
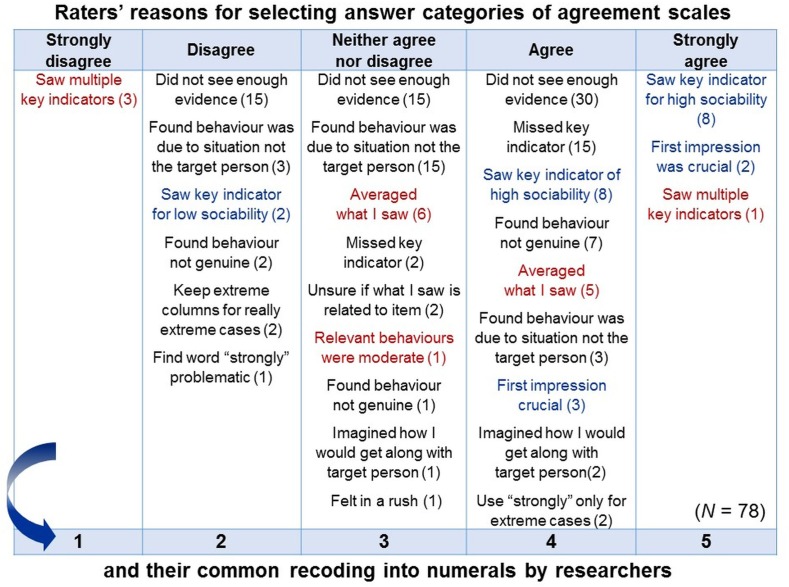
Raters’ interpretations of scale categories. Reasons provided by *N* = 78 raters for their ratings of a target person seen in a film on the BFI-10 item “… is outgoing, sociable”. Numbers in parentheses indicate absolute frequencies of reasons provided; multiple nominations possible.

But similar to item interpretations, raters’ interpretation and use of scale categories vary substantially within and between raters (Rosenbaum and Valsiner, 2011). When asked to judge a film protagonist’s personality on a five-stage agreement scale and to explain their choice of the answer category, 78 raters provided very different reasons (Figure 13 depicts reasons provided for the BFI-10 item “is outgoing, sociable” operationalizing the construct Extraversion; Uher, unpublished). Only 10.7% of all explanations indicated that raters considered and weighted various pieces of evidence as commonly assumed (highlighted in red). About 15% indicated that raters based their ratings on the occurrence of one single instance, such as a key indicator or their first impression (highlighted in blue), ignoring all other pieces of evidence available. Most explanations (67.7%) showed that raters found there was not enough evidence to make a judgment, that they missed key indicators, attributed the behaviors observed to the situation or found them not genuine, and thus not indicative of the target’s personality, among further reasons. But all raters had ticked a box.

The diversity of reasons for ticking rating scales and the triviality of many decisions that raters reported to have made shows that raters’ interpretations of scale categories vary considerably. Quite many interpretations do not refer to quantitative considerations at all. Moreover, raters assigned the same reasons to different categories, which have thus not distinct but overlapping meanings (Figure 13). This shows that raters do not interpret and use these scales in standardized ways, and that they do not apply fixed determinative assignment rules to indicate their judgments in the scale units. Neither object-dependence nor subject-independence can be established. But all this remains unknown because raters are commonly not asked to explain how they have generated their ratings.

#### Lack of Traceability of Intuitive Ratings Cannot Be Overcome by Converting Rating Scale Categories *post hoc* Into Numerals

**FIGURE 14:**
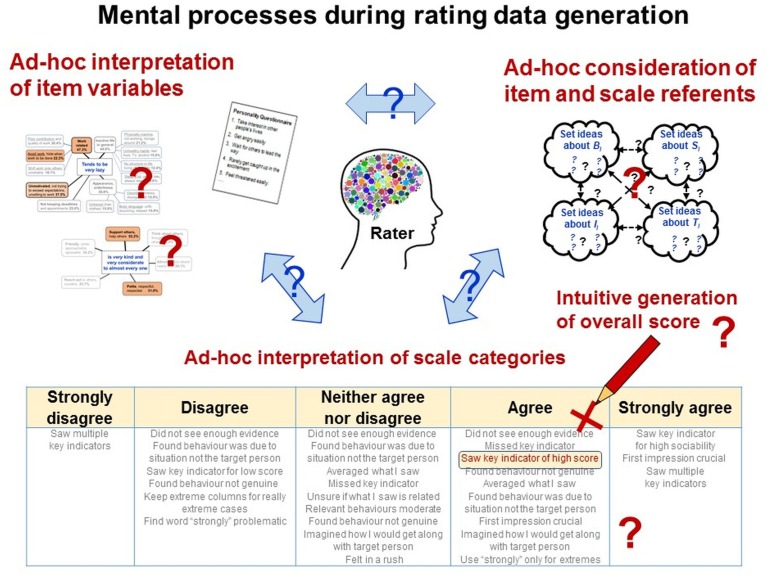
Mental processes involved in rating generation. Raters’ *ad hoc* interpretations of the rating items and scales, their ad hoc decisions about the actual objects of research as well as their formation of an overall judgment remain unknown.

Once raters have completed raw data generation, researchers commonly recode the (often lexically encoded) answer categories into numerals and treat these as numbers. This means that “have not seen enough evidence” can be recoded into the same numerical score as “missed a key indicator” or “found the behavior not genuine” (e.g., “4”). Likewise, “found behavior was due to situation not the target person” and “unsure if what I saw is related to the item” can be recoded into a higher Extraversion score for the target person (e.g., “3”) than “have seen a key indicator for low sociability” (e.g., “2”). Hence, the interrelations of the numbers into which researchers recode scale categories (e.g., order of magnitude) do not match the interrelations of the answer categories as raters have interpreted and used them (Figures 13, 14). Instead of constituting quantities of the quality specified in the item, raters’ interpretations of scale units rather constituted further *qualities* in themselves, such as ideas of the considered behaviors’ authenticity, relevance and situation-dependence.

**FIGURE 15:**
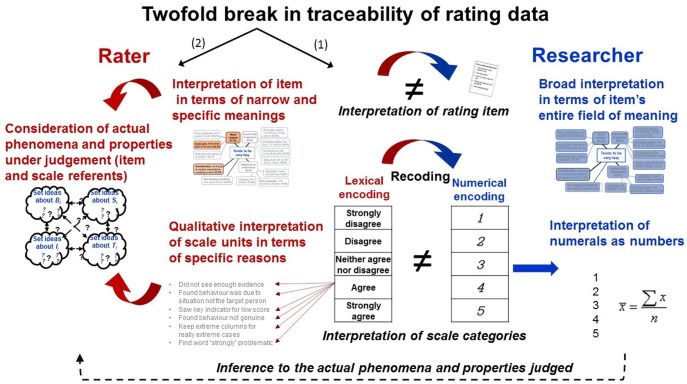
Twofold break in traceability. Researchers’ broad item interpretations and rigid recoding of answer categories into numerals interpreted as numbers entail shifts in interpretation that break the connections to (1) raters’ interpretations of items and scale units, and thus also to (2) raters’ perceptions and interpretations of the actual phenomena and properties that raters have considered and encoded in their ratings.

Summarizing, the requirement to generate data introquestively and long-term memory-based, the lack of information about the representational system under study and the constraint response format prevent that researchers come to know about how raters actually understand and use rating scales. This precludes intersubjective discussion about the interpretation of the data generated and thus establishing subject-independence. Instead, researchers commonly interpret rating data with regard to the meanings that *they themselves* assign to the item variables, supported by countless validation studies. But for any single rating, raters obviously do not consider the broad fields of meaning an item may generally have in their sociolinguistic community. Instead, for the specific case at hand, they construe specific meanings, which constitute only a fraction of the item’s overall field of meaning (Figures 14, 15).

Moreover, researchers interpret the units and values of rating data with regard to the meanings of numbers that *they themselves* assign to the scale categories. This recoding of units constitutes a conversion of information that, in itself, is based on well-documented and unbroken chains of comparisons from raters’ ticks on the scales, thus creating perfect traceability. But the quantifications thus-obtained cannot be traced to the referents (empirical relational system) that raters have aimed to encode in their ratings (symbolic relational system), thus also precluding the establishment of object-dependent data generation processes. Researchers’ rigid recoding of answer categories breaks the chain of traceability that could be established if raters’ judgment and encoding processes were systematically explored (Figures 14, 15).

## Conclusion

Application of the TPS-Paradigm’s metatheoretical and methodological frameworks opened up novel perspectives on methods of quantitative data generation in psychology and social sciences. They showed that concepts from metrology can be meaningfully applied even if the objects of research are abstract constructs. But they also revealed serious limitations of rating methods.

Psychological and social-science concepts of ‘measurement’ were not built on metrological theories and not meant to meet metrological criteria. But when ratings are treated as ‘quantitative’ data and subjected to statistical analysis, and when their results are used to make inferences on and decisions about individuals and real-world problems, then the generation of these numerical data must conform to the principles of scientific (metrological) measurement. In the times of replication crises and Big Data, these methodological mismatches can no longer be ignored.

### Quantitative Versus Qualitative Methods – An Inaccurate and Misleading Divide

The analyses showed that all quantitative methods presuppose qualitative categorizations because “quantities are *of* qualities” (Kaplan, 1964, p. 207). Objects of research can only be identified by their qualities. The common polarization of so-called ‘quantitative methods’ versus ‘qualitative methods’, reflects misconceptions of the measurement-theoretical foundations of scientific quantification.

Raters’ explanations of their scale responses revealed that they consider rating scale units not as quantitative but rather as qualitatively different categories. Researchers increasingly consider this, such as by reporting percentages of raters who ticked particular categories rather than calculating averages or medians over rigidly assigned numbers. Unlike rating methods, many so-called ‘qualitative methods’, feature operational structures to establish object-dependent data generation processes and traceable outcomes (Uher, 2018a). Qualitative data thus-generated can be used to derive *post hoc* ratio-scaled quantifications, such as by computing frequencies of the occurrences of key themes in textual data (e.g., content analysis; Flick, 2014). In summary, all methods inherently explore qualitative properties of their objects of research and only some of them *additionally* enable these qualitative properties to be quantified.

The concept of semiotic representations illuminates a further controversy underlying the qualitative-quantitative debate. So-called quantitative researchers (using rating methods) focus on the interrelations between the signs’ physical constituents (signifier; e.g., item statements) and their referents (e.g., target persons’ behaviors), whereas so-called qualitative researchers focus on the signifiers’ interrelations with the meanings (the signified) that particular persons construct for them (Figure 3). The former researchers tend to ignore the composite’s psychical constituent, the latter its referent (see similarly Bhaskar, 1994). This shows that the different epistemologies underlying so-called qualitative and quantitative methods are not *per se* incommensurate with one another. Rather, their proponents only focus on different aspects in the triadic relations inherent to semiotic representations. This metatheoretical concept will therefore be useful to help find common ground and develop integrative concepts and methodologies in the future.

### Assessments on Rating Scales Are Not Measurements—Rating Data do Not Constitute Scientific Quantifications

Not every quantification is an outcome of measurement. For valid inferences from quantifications generated to properties of the actual phenomena under study, measurement processes must be established that are object-dependent, producing results that are subject-independent and thus traceable.

Key to scientific measurement and quantification is standardization. But not any kind of standardization fulfills the necessary requirements. Standardized scale presentation, administration, instruction and scoring are fundamental to rating methods (Walsh and Betz, 2000). But they standardize only the format of data encoding, not the ways in which raters actually generate the data. Therefore, assessments do not constitute measurements and should not be labeled as such. The current use of the term measurement in psychology and social sciences largely constitutes a cross-disciplinary jingle fallacy (same term denotes different concepts; Thorndike, 1903), which creates misunderstandings and hampers exchange and development.

### Problematic Assumptions in Psychometrics

The numerals into which psychometricians rigidly recode raters’ ticks on the scales do not constitute measurement-based quantifications. Rasch analysis and conjoint measurement, often assumed to enable quantitative measurement with rating data (Borsboom and Mellenbergh, 2004; Michell, 2014), are only methods for modeling data *once they have been generated*. These methods show that rating data, *as recoded and interpreted by the researchers* (i.e., units interpreted as reflecting numbers, items as reflecting broad fields of meanings) can exhibit particular quantitative properties (e.g., additivity). But these properties are obtained through rigorous psychometric variable selection that align the data generation process to statistical assumptions rather than to properties of the actual objects of research, thus precluding object-dependence.

This entails a *twofold break in traceability in the triadic interactions involved in human-generated data generation*—first, to raters’ interpretation and use of the rating scales as methods, and second, to their perceptions and interpretations of the actual phenomena and properties under study. As a consequence, quantitative properties ascertained in psychometric analyses cannot be attributed to the actual referents of the raw data (e.g., target persons’ properties) as conceived by the raters who have generated these data (Figure 15).

### Consequences for the Replicability and Transparency of Data

The methodological problems involved in rating methods, especially the inability to establish traceable chains of information conversions from the objects of research to the outcomes of data generation, may constitute a major reason for the lack of replicability in psychology and social sciences not yet considered. “Robust measures”, often proposed as a solution to this problem (Carpenter, 2012; Yong, 2012; Asendorpf et al., 2013), are unlikely to be attained with rating-based quantifications. On the contrary, the standardisations implemented in rating methods may lead to systematic errors because consistency in data structure is achieved at the cost of data accuracy in terms of standardized and traceable relations to the actual phenomena under study and the ways in which they were quantified (see Hammersley, 2013).

### Consequences for the Validity and Utility of Data: Interpretability Presupposes Traceability

Nowadays, rating data can be generated quickly and at large scale (e.g., online-questionnaires; Buhrmester et al., 2011; Chandler and Paolacci, 2017; Buchanan and Scofield, 2018) producing floods of data—Big Data. But to answer research questions and to find solutions for real-world problems, scientists must eventually interpret the data produced. This article showed that, in the process of *data generation*, information must be converted from perceptions and conceptions of the study phenomena into signs. But in the process of *data interpretation*, information must be converted in the reverse direction from the signs back to conceptions and ideas about the actual phenomena under study. Such backward conversions of information may not be straightforwardly possible because signs, especially mathematical ones, can be abstracted, processed and changed in ways not applicable to the properties of the actual study phenomena (Brower, 1949; Trierweiler and Stricker, 1998), highlighting the importance of traceability not only in data generation but also in data analysis.

### Major Tasks Still Laying Ahead

As interpretations of rating scales are based on everyday knowledge with its fuzzy and flexible categories, any interpretation of rating data can appear plausible (Laucken, 1974). But the purpose of scientific measurement is to quantify phenomena in the real world—not to construe a possible match with data that can be generated even in absence of the persons, phenomena and properties under study. Therefore, traceability is a fundamental requirement for scientific quantification that should be implemented systematically also in the methods used to generate quantitative data in psychology and the social sciences. This article started to elaborate some principles by which this can be achieved.

Psychologists and social scientists must finally investigate how people actually understand and use rating scales to generate quantitative data in research and applied contexts. Exploring raters’ mental processes and the meanings they attribute to items and scale categories is key to specifying the representational systems underlying rating data, which, in many fields, make up much of the current empirical data basis.

## Author Contributions

The author confirms being the sole contributor of this work and has approved it for publication.

## Conflict of Interest Statement

The author declares that the research was conducted in the absence of any commercial or financial relationships that could be construed as a potential conflict of interest.

## Footnotes

1 The distinction between ‘data generated *with* persons’ versus ‘data generated *on* persons’ (frequently made in metrology) is irrelevant for the present analyses that focus on the processes by which persons generate data, no matter whether these data are about persons, non-human animals or objects.

2 researchonindividuals.org

3 This distinction is made in many languages (e.g., Dutch, French, German, Italian, and Russian) but not commonly in the English.

4 In the TPS-Paradigm, assumptions of extended mind are rejected because psychical phenomena in themselves are differentiated from their possible expression in behaviors and language, which form inherent parts of extended mind concepts (Clark and Chalmers, 1998; Logan, 2007).

5 Derived from the Latin *nunc ipsum* for at this very instant.

6 Derived from the Latin *intro* for in, within; and *quaerere* for to seek, enquire.

7 Derived from the Latin *extro* for beyond, outside.

8 In metrology, ordinal scaled data do not constitute quantifications (BIPM, 2006).

9 Not all introquestive methods are based on long-term memory recall. Further methods involve *nunc-ipsum introquestion* (e.g., thinking aloud methods) and *retro-introquestion* (e.g., diary methods; for details and criteria, see Uher, 2018a).
